# REMAS: a new regression model to identify alternative splicing events from exon array data

**DOI:** 10.1186/1471-2105-10-S1-S18

**Published:** 2009-01-30

**Authors:** Hao Zheng, Xingyi Hang, Ji Zhu, Minping Qian, Wubin Qu, Chenggang Zhang, Minghua Deng

**Affiliations:** 1 LMAM, School of Mathematical Sciences and Center for Theoretical Biology, Peking University, Beijing 100871, PR China; 2 Beijing Institute of Radiation Medicine, State Key Laboratory of Proteomics, Beijing 100850, PR China; 3 Department of Statistics, University of Michigan, Ann Arbor, MI 48109-1107, USA

## Abstract

**Background:**

Alternative splicing (AS) is an important regulatory mechanism for gene expression and protein diversity in eukaryotes. Previous studies have demonstrated that it can be causative for, or specific to splicing-related diseases. Understanding the regulation of AS will be helpful for diagnostic efforts and drug discoveries on those splicing-related diseases. As a novel exon-centric microarray platform, exon array enables a comprehensive analysis of AS by investigating the expression of known and predicted exons. Identifying of AS events from exon array has raised much attention, however, new and powerful algorithms for exon array data analysis are still absent till now.

**Results:**

Here, we considered identifying of AS events in the framework of variable selection and developed a regression method for AS detection (REMAS). Firstly, features of alternatively spliced exons were scaled by reasonably defined variables. Secondly, we designed a hierarchical model which can represent gene structure and transcriptional influence to exons, and the lasso type penalties were introduced in calculation because of huge variable size. Thirdly, an iterative two-step algorithm was developed to select alternatively spliced genes and exons. To avoid negative effects introduced by small sample size, we ranked genes as parameters indicating their AS capabilities in an iterative manner. After that, both simulation and real data evaluation showed that REMAS could efficiently identify potential AS events, some of which had been validated by RT-PCR or supported by literature evidence.

**Conclusion:**

As a new lasso regression algorithm based on hierarchical model, REMAS has been demonstrated as a reliable and effective method to identify AS events from exon array data.

## Background

Alternative splicing (AS) is an important regulatory mechanism in eukaryotes to increase proteome diversity by allowing the production of multiple isoforms from a single gene. It is one of the most extensive phenomena that account for complexity of molecular function through the combination of splice sites. Many AS events can modulate protein function and structure by gain or loss of domains coded by alternatively spliced exons [1]. At the same time, the disrupted code and machinery of splicing have roles in various diseases, e.g. cystic fibrosis, type I diabetes and myocardial infarction [2]. Therefore, genome-wide exploration of AS events will pave the way for future attempts to develop novel therapy strategies for diseases caused by AS [3].

Besides the assembly of cDNA sequences and expressed sequence tags (ESTs) to predict AS events [4], high-throughput microarray techniques had been widely used to identify AS events in genome-wide [5]. Clark *et al. *designed an oligonucleotide-spotted array probing at intron-exon junction to distinguish spliced RNA from unspliced ones and study the influences on splicing by eliminating the effects of splicing factors [6]. Yeakley *et al. *developed a novel bead-based fiber optic array with high sensitivity to perform a parallel analysis of mRNA isoforms in human [7]. Johnson *et al*. designed a splicing array monitoring exon-exon junctions of human RefSeq mRNAs in 52 tissues and cell lines [8]. Pan *et al. *combined information from six probes (half of them for exon-exon junctions and the rest for exon bodies) to quantitatively identify tissue-specific AS in mammalian cells [9]. Ule *et al. *customized a kind of microarray with probes targeting exon bodies and junctions to investigate the function of a neuron-specific splicing factor [10]. Conclusively, these microarrays are capable to distinguish the exon architecture of transcript variants. However, they are limited to interrogate predetermined exons and exon junctions with restricted resolution.

Affymetrix recently published a high density exon-centric microarray, GeneChip^@ ^Exon 1.0 ST Array, which covers a high density of exon regions with roughly 40 probes per gene and altogether over 5.5 million features on each array (see GeneChip^@ ^Exon Array Design Technical Note, Affymetrix). Exon array can identify AS events and uncover novel exons by probing all known and predicted exon regions. For the first time, both gene-level and exon-level expression can be studied in genome-wide with a single array, which promote understanding both transcription and splicing regulation.

Methods for detecting AS events from microarray data have been studied to address different platforms or analysis steps [11-13]. However, algorithm focusing on indentifying AS from Affymetrix exon array is still lacking. Since there are few substantially validated AS datasets for exon array, it is challenging to develop and evaluate an effective prediction algorithm with few false positives. "Splicing Index" (SI), a basic linear model for estimating changes of exon expression, is most widely used in identification of AS from exon array [14-16]. Xing *et al. *also introduced a novel probe selection strategy for gene signal estimation to eliminate opposite effects introduced by alternatively spliced exons[17]. A program named 'GeneBASE' was then developed based on the probe selection strategy and a probe-specific background correction procedure [18]. However, new and powerful methods to identify AS other than SI model are still necessary.

In this study, we utilized a shrinkage and selection strategy for linear regression based on improved "lasso" method[19] to select alternatively spliced genes and exons. Parameter and variable indicating splicing capability are defined to quantitatively scale the features of AS events. By controlling the splicing parameters in the regression model, AS events can be selected from numerous candidates and ranked by confidence. Simulations and real data evaluation suggest that REMAS is reliable and effective to identify AS events from exon array data.

## Methods

Firstly, we used a linear formula to model gene structure with exons. We suppose there are *K *genes on chip of exon array, and the *i*^th ^gene has *p*_*i *_exons. Logarithmic values of probe sets signals after normalization and estimation by PLIER (see Probe Logarithmic Intensity Error Estimation Technical Note, Affymetrix) algorithm are taken as expression of exon. "*exon*_*i*, *j*_" denotes expression of the *j*^th ^exon in the *i*^th ^gene. We define variable *x*_*i*, *j *_titled with 'AS Indicator' (ASI) as following:

(1) $$x_{i,j}=exon_{i,j}-median_{j\in \left\{1,\cdots p_{i}\right\}}\left\{exon_{i,j}\right\}$$

The intuition behind is that intensities of constitutive exons (relative to alternatively spliced exons) are strongly correlated to the overall gene expression. Thus ASI is considered as the expression difference between alternatively spliced exon and dominant constitutive exons and low-weighted effects introduced by AS can be reduced by median function to estimate a more accurate value of gene. Normally, the absolute value of ASI is close to zero for constitutive exon and much larger than zero for alternatively spliced exon.

We regard the class label as the response variable in our regression model, and denote it using y. A basic regression model can be applied to feature the relationship between ASI and *y*:

(2) $$\begin{array}{l} y=\beta_{0}+x_{1,1}\beta_{1,1}+\cdots +x_{1,p_{1}}\beta_{1,p_{1}}+\cdots \\ +x_{K,1}\beta_{K,1}+\cdots +x_{K,p_{K}}\beta_{K,p_{K}}+\varepsilon \end{array}$$

We primarily focus on the coefficients *β*_*i*, *j *_in formula (2). The larger the absolute value of coefficient *β*_*i*, *j*_, the stronger the potentials for AS events. Furthermore, we decompose *β*_*i*, *j *_into two parameters *α*_*i *_and *θ*_*i*, *j *_representing effects from gene level and exon level respectively as following:

(3) *β*_*i*, *j *_= *α*_*i*_·*θ*_*i*, *j*_

The parameter *α*_*i *_(*α*_*i *_> 0) is a positive real number measuring the regulatory influence to exon from gene level. The gene level regulation from alternatively spliced gene is different from constitutively spliced genes, so that affected exons can be inferred by the value distribution of *α*_*i*_. After then, a particular *θ*_*i*, *j*_, which is used to infer the influence from exon level indicating the alternatively spliced exon and its position in the gene. Based on this idea, formula (2) can be transformed to:

(4) $$y=\beta_{0}+\sum_{i=1}^{K}\alpha_{i}\cdot \sum_{j=1}^{p_{i}}x_{i,j}\theta_{i,j}+\varepsilon$$

Note that by location transformation, we can always assume that the predictors and the response have mean 0, so we can ignore the intercept in equation (4)

For real exon array data, the number of variables is quite huge (up to several millions), while the number of samples is small (usually less than 100). Therefore, the variable selection procedure is absolutely necessary for the regression model, which uses the theory of "lasso" for reference. The restraint for L_1 _norm is introduced to perform the variable selection. Regression in REMAS still has a good performance when sample size is much less than number of variables because of the L_1 _penalty. In practice, two parameters *t*_1 _and *t*_2 _are set as certain thresholds to restrict the following L_1 _constraints.

$$\sum_{i=1}^{K}\alpha_{i}<t_{1},\;\sum_{i=1}^{K}\sum_{j=1}^{p_{i}}\left|\theta_{i,j}\right|<t_{2}$$

If variables don't indicate AS events between samples, the corresponding coefficients would converge to zero. This procedure is equivalent to the minimization of the loss function below.

(5) $$L\left(\alpha ,\theta \right)=\sum_{l=1}^{n}{\left(y_{\left(l\right)}-\sum_{i=1}^{K}\alpha_{i}\cdot \sum_{j=1}^{p_{i}}x_{i,j,\left(l\right)}\theta_{i,j}\right)}^{2}+\mu \cdot \sum_{i=1}^{K}\alpha_{i}+\upsilon \sum_{i=1}^{K}\sum_{j=1}^{p_{i}}\left|\theta_{i,j}\right|$$

*μ *and *ν *are two fine-tuning parameters in the formula above, where *μ *controls the estimation of parameter *α*_*i *_for gene level while *ν *controls the estimation of parameter *θ*_*i*, *j *_for exon level. These hierarchical controls are not complicated to tune in practice because the two parameters *μ *and *ν *can be simplified into one parameter as *λ *= *μ*·*ν*. Subsequently, we can show that minimization of equation (5) is equivalent to the minimization of following loss function,

(6) $$L\left(\alpha ,\theta \right)=\sum_{l=1}^{n}{\left(y_{\left(l\right)}-\sum_{i=1}^{K}\alpha_{i}\cdot \sum_{j=1}^{p_{i}}x_{i,j,\left(l\right)}\theta_{i,j}\right)}^{2}+\sum_{i=1}^{K}\alpha_{i}+\lambda \sum_{i=1}^{K}\sum_{j=1}^{p_{i}}\left|\theta_{i,j}\right|$$

Here the equivalence is used to mean that the final fitted *β*_i, j _from equation (5) and equation (6) are the same, although they may corresponding to different *α*_i _and *θ*_i, j _. Thus we only need to tune one parameter *λ*. In practice, the parameter *λ *is set in advance before the optimization. We used a cross-validation method to obtain a suitable *λ *on performance.

A two-step iterative way is applied to estimate the parameter *α *and *θ *in the regression. For example, *θ *is initialized as constant to estimate *α*; then *α *is set as constant and *θ *is updated by minimizing the loss function in the same way. Actually, each procedure in the iteration is a typical 'lasso' problem as described in classical "lasso" algorithm[19]. Since some true AS events can't be selected out when the sample size is small, we introduce a complementary improvement by ranking the selected genes based on the parameter *α*. Genes with high potential of AS are selected first and then classified into group I, and other genes are temporally classified into group II. After removing exons of group I gene, the iterative procedure is carried out to sort the rest of genes, and a most potent gene can be selected out from group II again. After the iterative selection, genes in group I (alternatively spliced genes) can be globally ranked by the parameter *α*. It is accepted that genes ranking high are more potential to undergo AS.

## Results

In order to evaluate the accuracy and sensitivity of REMAS, simulation covering a diversity of sample sizes, AS types and regulatory patterns are performed and a real exon array dataset interpreting colon cancer was used to do the test [16]. Alternatively spliced exons predicted by the above mentioned SI algorithm are compared with prediction of REMAS.

### Evaluation on simulated data

Previous study has reported that probesets targeting those constitutive exons are strongly correlated across various samples[17]. Multiple normal distributions with proper covariance were used to simulate these constitutive exons as follows.

$$\left(\begin{array}{ccccc} 1 & 0.8 & 0.8 & \cdots & 0.8\\ 0.8 & 1 & 0.8 & \cdots & 0.8\\ 0.8 & 0.8 & 1 & \cdots & 0.8\\ \vdots & \vdots & \vdots & & \vdots \\ 0.8 & 0.8 & 0.8 & \cdots & 1 \end{array}\right)$$

Three different simulations were designed to evaluate the performance of REMAS on different scenarios. For each evaluation, two sources of samples (*e.g. *treatment group and control group) are designed for comparison. There are 50 genes containing 10 exons each in each sample.

#### Simulation 1

As shown in Table 1, we designed 100 samples for both groups, and 4 genes are alternatively spliced genes in each sample. For alternatively spliced gene 1 and 2, exon 5 and exon 6 were defined as "cassette" exons (a kind of splicing type with a form of exon skipping or inclusion) in treatment group. For alternatively spliced gene 3 and 4, exon 5 and exon 6 were simulated as mutually exclusive exons, in the sense that only one exon is included in the mature mRNA in a tandem array of alternative exons [20]. These splicing patterns were simulated by different normal distributions for comparison (see Table 1). However, constitutive exons are uniformly set as highly correlated normal distributions.

**Table 1:** Design of simulation 1 for evaluation

Gene ID	Treatment Group	Control Group
1	Exon 5~N (0, 1)	All exons ~N (2.5,1), correlated
	Exon 6~N (0, 1)	
	Others ~N (2.5, 1), correlated	
2	Exon 5~N (0, 1)	All exons ~N (1.5,1), correlated
	Exon 6~N (0, 1)	
	Others ~N (1.5, 1), correlated	
3	Exon 5~N (2.5, 1)	Exon 5~N (0, 1)
	Exon 6~N (0, 1)	Exon 6~N (2.5, 1)
	Others ~N (2.5, 1), correlated	Others ~N (2.5, 1), correlated
4	Exon 5~N (1.5, 1)	Exon 5~N (0, 1)
	Exon 6~N (0, 1)	Exon 6~N (1.5, 1)
	Others ~N (1.5, 1), correlated	Others ~N (1.5, 1), correlated

Exon 5 and Exon 6 are simulated as cassette exons in alternatively spliced gene 1 and 2, while they are defined as mutually exclusive exons in alternatively spliced gene 3 and 4.

**Figure 1:**
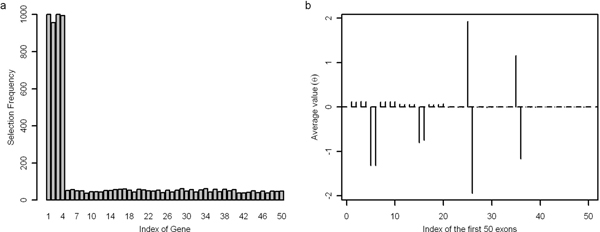
**Results of simulation 1**. Panel 'a' shows frequencies of selection by REMAS for the 50 genes in simulation 1. Panel 'b' shows distribution of average values of *θ *for the first 50 exons in simulation 1. The total number of selection is 1000.

Given a specific *λ*, we performed a five-fold cross-validation to measure the accuracy of prediction. The optimal *λ *= 0.5 was concluded from 500 different runs. For the tuning parameter *λ *= 0.5, we simulated 1000 times to select genes and exons undergoing AS. As illustrated in Figure 1 Results of simulation 1a, four simulated alternatively spliced genes have extremely high frequencies to be selected out, while other 46 genes are on the contrary. Obviously, REMAS is an effective discriminator for selection of alternatively spliced genes. Figure 1b shows the property of *θ *for the first 50 exons in selection. The left were ignored in the panel because they have uniform expression as constitutive exons. Y-axis describes the average values of *θ*_*i*, *j *_for 1000 times of selection. It is concluded that the absolute value of average *θ*_*i*, *j *_for alternatively spliced exons are significantly higher than constitutive exons. Average value of *θ*_*i*, *j*_proportionally changes following the power of normal distribution (see Figure 1b and Table 1). Therefore, the ascending or descending trend of *θ*_*i*, *j *_effectively represents exon inclusion or exon skipping respectively.

Flocks in Figure 1b are small positive values rose as false positive *θ *values in alternatively spliced gene 1 and 2. They are caused by the randomness of simulations, because the expression of alternatively spliced exons is possible to be the median of expression of all exons in the same gene. At the same time, other constitutive exons will be considered as alternatively spliced exons by error. *θ *values of these flocks are higher than those of fake alternatively spliced exons, but much less than true positives. As to alternatively spliced gene 3 and 4 with mutually exclusive splicing, positive and negative *θ *values exactly represent the pre-defined splicing pattern. Opposite *θ *values compromised each other so that the average of *θ *is close to zero.

#### Simulation 2

**Figure 2:**
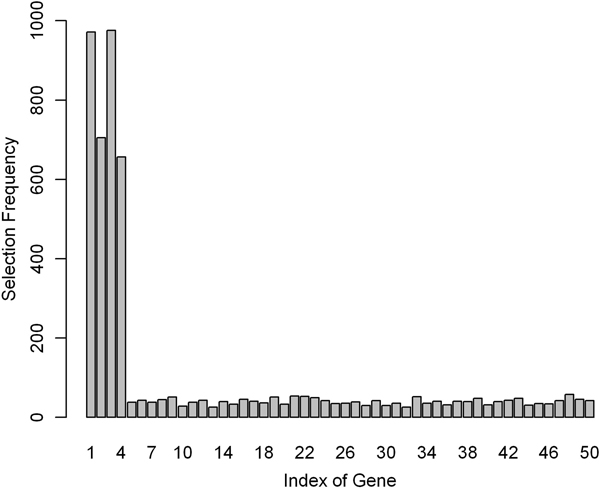
**Selection frequency in the first iteration in simulation 2**. Frequencies of the 50 genes selected by REMAS in simulation 2 are illustrated. The total number of selection is 1000.

This simulation serves as a supplement to simulation 1 to evaluate the performance of REMAS on small size samples. Most of the conditions were preserved except that the sample size was reduced to 10 for each group. The same procedure was repeated following simulation 1. REMAS still showed high stability in selecting alternatively spliced gene 1 and 3, but the rate of successfully selection of alternatively spliced gene 2 and 4 decreased (See Figure 2). Optimized *λ *is set as 0.001 in five-fold cross-validation at this simulation.

**Figure 3:**
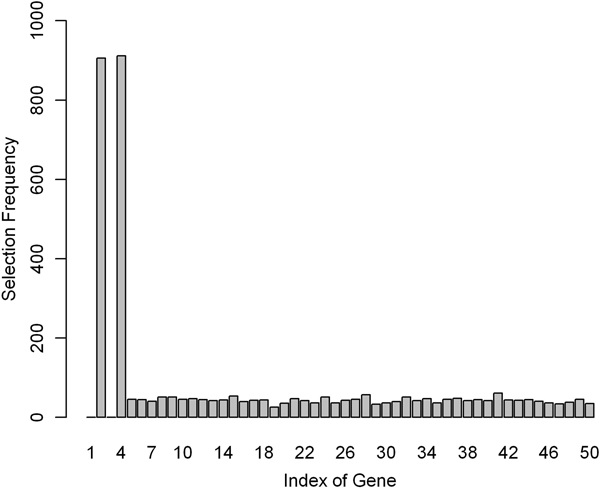
**Selection frequency in the second iteration in simulation 2**. Frequencies of the 48 genes selected by REMAS in simulation 2 after removing alternatively spliced gene 1 and 3. The total number of selection is 1000.

In order to make up the loss of capability, an iterative selection strategy was introduced to improve the sensitivity of REMAS. The most significant alternatively spliced gene was selected in iteration, and the other genes and exons remained for the next selection. For example, alternatively spliced gene 1 and 3 and their exons are removed in simulation 2 after the first iteration. As shown in Figure 3, gene 2 and 4 are significantly selected out in the second round of simulation 2. Optimized *λ *equals to 0.5 in five-fold cross-validation. Conclusively, this strategy provides an effective measurement to maximally reduce the negative effect of small sample size.

#### Simulation 3

This simulation was designed to describe the coupled regulation of transcription and splicing when AS events arose in the differentially expressed genes. It is challenging for REMAS to accurately predict AS because complex regulations result in a complicated data distribution. Simulated exons are identical with simulation 1, while the overall gene expression is set differently between two groups by controlling the powers of normal distribution (see Table 2).

**Table 2:** Design of simulation 3 for evaluation

Gene ID	Treatment Group	Control Group
1	Exon 5~N (0, 1)	All exons ~N (1.5,1), correlated
	Exon 6~N (0, 1)	
	Others ~N (2.5, 1), correlated	
2	Exon 5~N (0, 1)	All exons ~N (2.5,1), correlated
	Exon 6~N (0, 1)	
	Others ~N (1.5, 1), correlated	
3	Exon 5~N (2.5, 1)	Exon 5~N (0, 1)
	Exon 6~N (0, 1)	Exon 6~N (1.5, 1)
	Others ~N (2.5, 1), correlated	Others ~N (1.5, 1), correlated
4	Exon 5~N (1.5, 1)	Exon 5~N (0, 1)
	Exon 6~N (0, 1)	Exon 6~N (2.5, 1)
	Others ~N (1.5, 1), correlated	Others ~N (2.5, 1), correlated

The design of alternatively spliced exons is same with simulation 1, but the power of normal distribution is different between two sample groups to simulate the differential expression in gene level.

**Figure 4:**
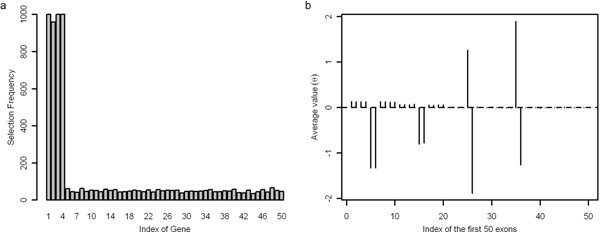
**Results of simulation 3**. Panel 'a' shows frequencies of selection by REMAS for the 50 genes in simulation 3. Panel 'b' shows distribution of average values of *θ *for the first 50 exons in simulation 3. The total number of selection is 1000.

Taken optimized *λ *= 0.5 in five-fold cross-validation, simulation was repeated for 1000 times to select alternatively spliced genes and exons. As shown in Figure 4a, four simulated genes undergoing AS could be selected robustly every time. Figure 4b represents the property of *θ *for the first 50 exons selected. The distribution of *θ *is same with property of *θ *in simulation 1 as illustrated in Figure 1. These results demonstrated that REMAS is robust enough to identify AS even in condition of sophisticated regulations.

### Evaluation on real exon array data

A public available human colon cancer data including 20 paired healthy and tumour samples were used to evaluate the performance of REMAS on real exon array data. Gardina *et al. *had analyzed the data based on SI algorithm and 189 putative AS events were predicted. Among them, 45 AS events have been validated by RT-PCR or literatures [16].

**Figure 5:**
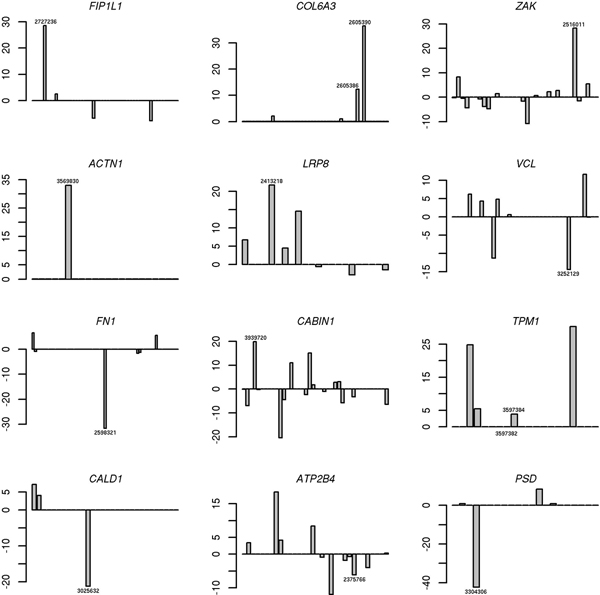
***θ *values for different exons in some genes which are identified by RT-PCR or supported by other literatures**. These 12 genes are not only identified by REMAS but also by SI algorithm. Importantly, they have been validated by RT-PCR or supported by literatures. The Affymetrix probeset ID (7-digit numbers) indicates the position of alternatively spliced exon in the target gene.

Probesets of exon array can be classified into three types according to their primary source and confidence (GeneChip^@ ^Exon Array Design Technical Note, Affymetrix). "Core" probesets are supported by well-curated mRNA sequences of RefSeq [21] and some other databases. "Extended" and "full" probesets are designed based on either low quality or predicted sequences. To minimize the influence of noises, only "Core" probesets were selected for evaluation. Selected with REMAS, alternatively spliced genes were ranked by values of *α*. There are 57 overlapping alternatively spliced genes between top 500 genes identified by REMAS and predicted by SI algorithm. Among the top ten genes in our ranking list, four of them have been validated by RT-PCR. It is also remarkable that 20 of 57 genes are validated by RT-PCR or supported by literatures (see Table 3). Furthermore, alternatively spliced exons were detected by *θ *to confirm their positions in gene structure. Twelve genes from Table 3 were selected to show distribution of *θ *values for exons along the gene (see Figure 5), which demonstrates that REMAS can detect alternatively spliced genes and exons effectively. For example, the 4^th ^and 6^th ^exon of *COL6A3 *gene are cassette exons which have been validated by RT-PCR. The *θ *values for both probeset 2605390 (Affymetrix probeset ID) targeting the 4^th ^exon and probeset 2605386 targeting the 6^th ^exon are significantly prominent in our results.

**Table 3:** List of identified alternatively spliced genes by REMAS validated by RT-PCR or supported by the literature

ID	Transcript cluster ID	Gene Symbol	Ranks
1	2425756	COL11A1	5
2	2727226	FIP1L1	11
3	2605321	COL6A3	18
4	3604147	KIAA1199	24
5	2515933	ZAK	28
6	3049522	TENS3	29
7	2907671	PTK7	77
8	3569814	ACTN1	120
9	2413203	LRP8	134
10	3252071	VCL	148
11	2598261	FN1	190
12	3939707	CABIN1	193
13	2712236	MUC4	212
14	3597338	TPM1	231
15	3735151	ITGB4	287
16	3025545	CALD1	307
17	2375706	ATP2B4	335
18	3304301	PSD	354
19	3694657	CDH11	418
20	3252036	PLAU	480

## Discussion

We developed an improved regression model named REMAS to select alternatively spliced genes and exons from exon array data. Both simulation and real data analysis indicate that REMAS has convincing capability in identification of AS events.

Although many splicing events deal with multiple exons, the linear SI algorithm ignores relationships between exons and identifies alternatively spliced exons individually. For example, mutually exclusive exon is one of the common AS patterns in eukaryotes. Regarding gene as an assembly of exons, REMAS can select those correlated exons in alternatively spliced gene in a single iteration (see results of simulation and Figure 5). Furthermore, REMAS can rank genes according to their potentials for AS. The ranking is also considered as an important confident index of prediction reliability.

Limitations also should be paid attention to our method. Here we only focus on the linear regression model. It is also possible to train a logistic regression model and perform the variable selection in the similar way. However, the computation will be much heavy than linear regression. Up to now, we cannot address a reasonable convergent threshold for AS detection in real data, because it is difficult to estimate how many AS events occur in samples in prior. When we are preparing our manuscript, Xing *et al. *recently published a new method detecting AS from exon array data with a high validation rate by RT-PCR[22]. The comparison between REMAS and this method and evaluation with their validated data will be addressed in our future works.

## Conclusion

AS is difficult to be validated because genes undergoing alternate splicing always express in specific conditions. Therefore, well-validated exon array datasets are very important for developing efficient methods. In despite of these facts, REMAS is a valuable choice for identifying AS events from exon array data with good performance and some unique advantages.

## Competing interests

The authors declare that they have no competing interests.

## Authors' contributions

HZ constructed the model and drafted the paper. XYH performed the simulation and drafted the manuscript. JZ, MPQ and WBQ performed some of the analysis and helped draft the manuscript. CGZ instigated and guided the research project and proof-read the manuscript. MHD supervised the analysis, figure preparation and finalized the manuscript. All authors read and approved the manuscript.
